# Genomic analysis of early-stage lung cancer reveals a role for *TP53* mutations in distant metastasis

**DOI:** 10.1038/s41598-022-21448-1

**Published:** 2022-11-09

**Authors:** Debra Van Egeren, Khushi Kohli, Jeremy L. Warner, Philippe L. Bedard, Gregory Riely, Eva Lepisto, Deborah Schrag, Michele LeNoue-Newton, Paul Catalano, Kenneth L. Kehl, Franziska Michor, for the AACR Project GENIE Consortium represented by Shawn Sweeney, Michael Fiandalo, Margaret Foti, Yekaterina Khotskaya, Jocelyn Lee, Nicole Peters, Shawn Sweeney, Jean Abraham, James D. Brenton, Carlos Caldas, Gary Doherty, Birgit Nimmervoll, Karen Pinilla, Jose-Ezequiel Martin, Oscar M. Rueda, Stephen-John Sammut, Dilrini Silva, Kajia Cao, Allison P. Heath, Marilyn Li, Jena Lilly, Suzanne MacFarland, John M. Maris, Jennifer L. Mason, Allison M. Morgan, Adam Resnick, Mark Welsh, Yuankun Zhu, Bruce Johnson, Yvonne Li, Lynette Sholl, Ron Beaudoin, Roshni Biswas, Ethan Cerami, Oya Cushing, Deepa Dand, Matthew Ducar, Alexander Gusev, William C. Hahn, Kevin Haigis, Michael Hassett, Katherine A. Janeway, Pasi Jänne, Arundhati Jawale, Jason Johnson, Kenneth L. Kehl, Priti Kumari, Valerie Laucks, Eva Lepisto, Neal Lindeman, James Lindsay, Amanda Lueders, Laura Macconaill, Monica Manam, Tali Mazor, Diana Miller, Ashley Newcomb, John Orechia, Andrea Ovalle, Asha Postle, Daniel Quinn, Brendan Reardon, Barrett Rollins, Priyanka Shivdasani, Angela Tramontano, Eliezer Van Allen, Stephen C. Van Nostrand, Jonathan Bell, Michael B. Datto, Michelle Green, Chris Hubbard, Shannon J. McCall, Niharika B. Mettu, John H. Strickler, Fabrice Andre, Benjamin Besse, Marc Deloger, Semih Dogan, Antoine Italiano, Yohann Loriot, Lacroix Ludovic, Stefan Michels, Jean Scoazec, Alicia Tran-Dien, Gilles Vassal, Christopher E. Freeman, Susan J. Hsiao, Matthew Ingham, Jiuhong Pang, Raul Rabadan, Lira Camille Roman, Richard Carvajal, Raymond DuBois, Maria E. Arcila, Ryma Benayed, Michael F. Berger, Marufur Bhuiya, A. Rose Brannon, Samantha Brown, Debyani Chakravarty, Cynthia Chu, Ino de Bruijn, Jesse Galle, Jianjiong Gao, Stu Gardos, Benjamin Gross, Ritika Kundra, Andrew L. Kung, Marc Ladanyi, Jessica A. Lavery, Xiang Li, Aaron Lisman, Brooke Mastrogiacomo, Caroline McCarthy, Chelsea Nichols, Angelica Ochoa, Katherine S. Panageas, John Philip, Shirin Pillai, Gregory J. Riely, Hira Rizvi, Julia Rudolph, Charles L. Sawyers, Deborah Schrag, Nikolaus Schultz, Julian Schwartz, Robert Sheridan, David Solit, Avery Wang, Manda Wilson, Ahmet Zehir, Hongxin Zhang, Gaofei Zhao, Lailah Ahmed, Philippe L. Bedard, Jeffrey P. Bruce, Helen Chow, Sophie Cooke, Samantha Del Rossi, Sam Felicen, Sevan Hakgor, Prasanna Jagannathan, Suzanne Kamel-Reid, Geeta Krishna, Natasha Leighl, Zhibin Lu, Alisha Nguyen, Leslie Oldfield, Demi Plagianakos, Trevor J. Pugh, Alisha Rizvi, Peter Sabatini, Elizabeth Shah, Nitthusha Singaravelan, Lillian Siu, Gunjan Srivastava, Natalie Stickle, Tracy Stockley, Marian Tang, Carlos Virtaenen, Stuart Watt, Celeste Yu, Brady Bernard, Carlo Bifulco, Julie L. Cramer, Soohee Lee, Brian Piening, Sheila Reynolds, Joseph Slagel, Paul Tittel, Walter Urba, Jake VanCampen, Roshanthi Weerasinghe, Alyssa Acebedo, Justin Guinney, Xindi Guo, Haley Hunter-Zinck, Thomas Yu, Kristen Dang, Valsamo Anagnostou, Alexander Baras, Julie Brahmer, Christopher Gocke, Robert B. Scharpf, Jessica Tao, Victor E. Velculescu, Shlece Alexander, Neil Bailey, Philip Gold, Mariska Bierkens, Jan de Graaf, Jan Hudeček, Gerrit A. Meijer, Kim Monkhorst, Kris G. Samsom, Joyce Sanders, Gabe Sonke, Jelle ten Hoeve, Tony van de Velde, José van den Berg, Emile Voest, George Steinhardt, Sabah Kadri, Wanjari Pankhuri, Peng Wang, Jeremy Segal, Christine Moung, Carlos Espinosa-Mendez, Henry J. Martell, Courtney Onodera, Ana Quintanar Alfaro, E. Alejandro Sweet-Cordero, Eric Talevich, Michelle Turski, Laura Van’t Veer, Amanda Wren, Susana Aguilar, Rodrigo Dienstmann, Francesco Mancuso, Paolo Nuciforo, Josep Tabernero, Cristina Viaplana, Ana Vivancos, Ingrid Anderson, Sandip Chaugai, Joseph Coco, Daniel Fabbri, Doug Johnson, Leigh Jones, Xuanyi Li, Christine Lovly, Sanjay Mishra, Kathleen Mittendorf, Li Wen, Yuanchu James Yang, Chen Ye, Marilyn Holt, Michele L. LeNoue-Newton, Christine M. Micheel, Ben H. Park, Samuel M. Rubinstein, Thomas Stricker, Lucy Wang, Jeremy Warner, Meijian Guan, Guangxu Jin, Liang Liu, Umit Topaloglu, Cetin Urtis, Wei Zhang, Michael D’Eletto, Stephen Hutchison, Janina Longtine, Zenta Walther

**Affiliations:** 1 Department of Data Science, Dana-Farber Cancer Institute, Boston, MA USA; 2 Department of Systems Biology, Harvard Medical School, Boston, MA USA; 3 Stem Cell Program, Boston Children’s Hospital, Boston, MA USA; 4 Department of Medicine, Vanderbilt University, Nashville, TN USA; 5 Department of Biomedical Informatics, Vanderbilt University, Nashville, TN USA; 6 Department of Medicine, University of Toronto, Toronto, ON Canada; 7 Department of Medicine, Memorial Sloan Kettering Cancer Center, New York, NY USA; 8 Department of Medical Oncology, Dana-Farber Cancer Institute, Boston, MA USA; 9 Vanderbilt-Ingram Cancer Center, Vanderbilt University Medical Center, Nashville, TN USA; 10 Department of Stem Cell and Regenerative Biology, Harvard University, Cambridge, MA USA; 11 Broad Institute of MIT and Harvard, Cambridge, MA USA; 12 Department of Biostatistics, Harvard T.H. Chan School of Public Health, Boston, MA USA; 13 The Center for Cancer Evolution, Dana-Farber Cancer Institute, Boston, MA USA; 14 The Ludwig Center at Harvard, Boston, MA USA; 15 Department of Medicine, Weill Cornell Medicine, New York, NY USA; 16 Present Address: Multiple Myeloma Research Foundation, Norwalk, CT USA; 17 American Association for Cancer Research, Philadelphia, PA USA; 18 Cancer Research UK Cambridge Centre, Cambridge, UK; 19 Children’s Hospital of Philadelphia, Philadelphia, PA USA; 20 Duke University (Duke Cancer Institute), Durham, NC USA; 21 Gustave Roussy Cancer Campus, Paris-Villejuif, France; 22 Herbert Irving Comprehensive Cancer Center, Columbia University, New York, NY USA; 23 Medical University of South Carolina, Charleston, SC USA; 24 Princess Margaret Cancer Centre, Toronto, ON Canada; 25 Providence Cancer Institute, Portland, OR USA; 26 Sage Bionetworks, Seattle, WA USA; 27 Sidney Kimmel Comprehensive Cancer Center at Johns Hopkins, Baltimore, MD USA; 28 Swedish Cancer Institute, Seattle, WA USA; 29 The Netherlands Cancer Institute, Amsterdam, Utrecht, The Netherlands; 30 The University of Chicago Comprehensive Cancer Center, Chicago, IL USA; 31 Diller Family Comprehensive Cancer Center), University of California-San Francisco (UCSF Helen, San Francisco, CA USA; 32 Vall d’ Hebron Institute of Oncology, Barcelona, Spain; 33 Vanderbilt-Ingram Cancer Center, Nashville, TN USA; 34 Wake Forest University Health Sciences (Wake Forest Baptist Medical Center), Winston-Salem, NC USA; 35 Yale University (Yale Cancer Center), New Haven, CT USA

**Keywords:** Cancer genetics, Statistics

## Abstract

Patients with non-small cell lung cancer (NSCLC) who have distant metastases have a poor prognosis. To determine which genomic factors of the primary tumor are associated with metastasis, we analyzed data from 759 patients originally diagnosed with stage I–III NSCLC as part of the AACR Project GENIE Biopharma Collaborative consortium. We found that *TP53* mutations were significantly associated with the development of new distant metastases. *TP53* mutations were also more prevalent in patients with a history of smoking, suggesting that these patients may be at increased risk for distant metastasis. Our results suggest that additional investigation of the optimal management of patients with early-stage NSCLC harboring *TP53* mutations at diagnosis is warranted in light of their higher likelihood of developing new distant metastases.

## Introduction

Distant metastasis in non-small-cell lung cancer (NSCLC) is associated with a poor survival of only 6% at 5 years after primary diagnosis^1^. About 50% of patients present with distant metastases at the time of diagnosis (i.e., Stage IV)^2^, and ~ 34% of patients diagnosed with stage I-II disease develop metastases five years after diagnosis^3^. While some studies suggest that specific mutations (e.g., in *EGFR*) increase the risk of distant metastasis^4^, other results indicate that these mutations do not significantly affect metastasis development^5^. To further investigate this question, we performed a retrospective analysis of 759 patients with stage I-III NSCLC who underwent targeted sequencing of their primary tumors as part of the AACR Project GENIE BPC NSCLC v2.1-consortium dataset^6^ to determine if specific mutations and copy number alterations common in NSCLC are associated with metastasis to distant sites.

**Figure 1:**
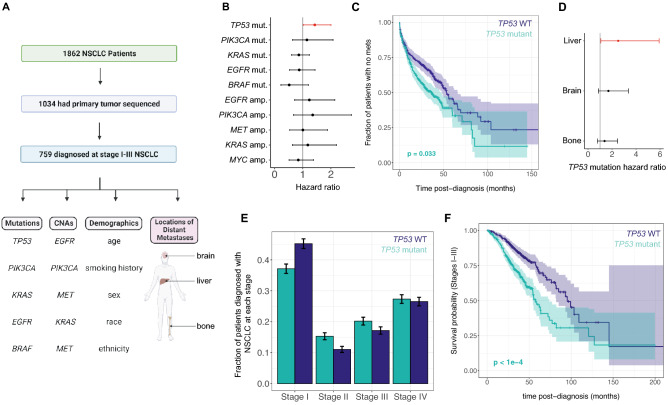
*TP53* mutations are significantly associated with the development of distant metastases after diagnosis in early-stage NSCLC. (**A**) Overview of study design. (**B**) Cox regression hazard ratios of each mutation and copy number alteration analyzed, with significant results (α = 0.05) in red. Error bars are Bonferroni-adjusted 95% confidence intervals. (**C**) Kaplan–Meier curves showing time to first distant metastasis among patients with early-stage disease, stratified by *TP53* mutation status in the primary tumor. Error bars are 95% confidence intervals. (**D**) Cox regression hazard ratios for *TP53* mutation for metastasis to individual sites, with significant results (α = 0.05) in red. Error bars are Bonferroni-adjusted 95% confidence intervals. (**E**) Fraction of patients diagnosed at each stage, stratified by *TP53* mutation status. Error bars denote 95% confidence intervals. (**F**) Kaplan–Meier curves showing overall survival probability stratified by *TP53* mutation status, for patients diagnosed with stage I-III disease. Error bars are 95% confidence intervals. Colors for all panels denote primary tumor *TP53* mutation status (dark blue: mutant, teal: wild-type). In all Cox regressions, we incorporated age, sex, race, ethnicity, smoking history, stage at diagnosis, and 10 total mutations/copy number alterations as covariates (Methods).

We used multivariate Cox proportional hazards models to quantify the association between common genomic alterations in the primary tumor and the rate of developing distant metastases in NSCLC patients diagnosed with local or locally advanced disease (stages I-IIIB; Fig. 1A, Supplementary Table 1, Methods). We investigated associations between nonsynonymous mutations in 5 of the most commonly mutated genes in NSCLC (*TP53, KRAS, EGFR, BRAF, PIK3CA*) and copy number changes in 5 of the most commonly amplified genes (*EGFR, PIK3CA, MET, KRAS, FGFR1*) and the likelihood of developing metastases. We found that *TP53* mutations were associated with a significantly increased rate of developing metastases to any distant site after diagnosis (Fig. 1B,C; HR = 1.43, HR 95% CI 1.09–2.90, *p* = 0.033, Wald’s Test with Benjamini-Hochberg (BH) adjustment for multiple hypothesis testing).

We also investigated associations between these mutations and CNAs and the development of metastases to specific distant sites individually (Fig. 1D) and found that *TP53* mutations were associated with a significantly increased rate of metastasis to the liver (HR = 2.51, HR 95% CI 1.07–5.93, BH-adjusted *p* = 0.026, Wald’s Test). However, no significant associations between any genomic alterations and the metastasis rate to brain or bone specifically were observed (Supplementary Fig. 1). We found that *TP53* mutation status was not significantly associated with NSCLC stage at diagnosis (*p* = 0.21, χ^2^ test) (Fig. 1E), but was significantly associated with reduced overall survival in patients diagnosed with stage I-III NSCLC (Fig. 1F and Supplementary Fig. 2; HR = 1.97, HR 95% CI 1.45–2.66, *p* < 1e-04, Wald’s test).

**Figure 2:**
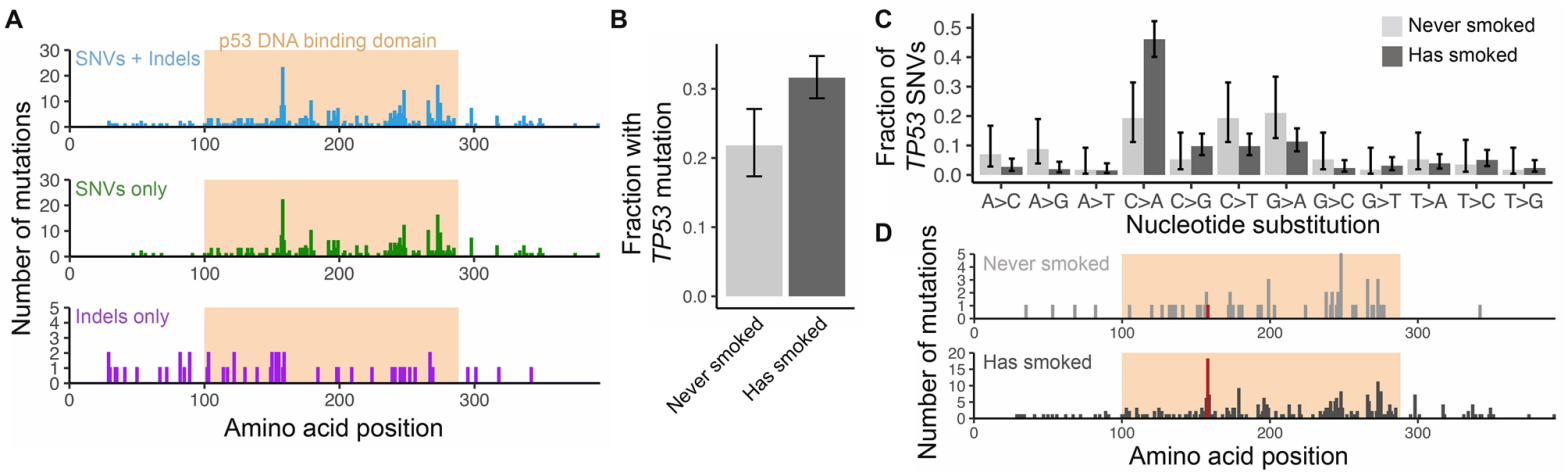
*TP53* SNVs are found in the DNA binding domain and are associated with smoking. (**A**) Location of nonsynonymous SNVs and/or frameshift indels in the *TP53* gene in primary tumor samples from stage I-IV NSCLC patients. The location of the p53 DNA binding domain is shown as an orange shaded region. (**B**) Fraction of patients with a *TP53* mutation, stratified by smoking history. (**C)** Frequency of specific nonsynonymous single nucleotide substitutions in *TP53* in patients without a history of smoking (light grey) and patients with a history of smoking (dark grey). In (**B**) and (**C**), error bars denote 95% Bayesian credible intervals. (**D**) Location of nonsynonymous SNVs and frameshift indels in smokers and nonsmokers, with amino acid position 158 highlighted in red.

Given the prognostic significance of *TP53* mutations in NSCLC, we analyzed the location and identity of *TP53* mutations found in primary tumors using an expanded cohort of 1,034 patients with stage I-IV disease (Methods). *TP53* mutations in cancer have previously been shown to occur mostly in the DNA binding domain^7,8^, suggesting that these mutations are likely to impair protein function. Of the 331 patients in our cohort with nonsynonymous point mutations or indels in *TP53*, 285 had mutations localized within the p53 DNA binding domain, most of which are single nucleotide substitutions (Fig. 2A). However, the splice site or frameshift insertions or deletions (n = 52 mutations) were more evenly spread throughout the coding sequence, likely because these mutations have a greater impact on protein function regardless of location.

We also found that *TP53* mutations were enriched in patients with a smoking history (*p* = 0.0023, χ^2^ test; odds ratio 1.66; Fig. 2B). Single nucleotide substitutions in *TP53* in smokers had a significantly different pattern of base substitutions than nonsmokers, with a higher rate of C > A substitutions found in smokers (Fig. 2C). This pattern is similar to the mutational signature associated with tobacco smoking in cancers of the lung and larynx^9^. The different mutational processes active in smokers and never-smokers were shown to result in differences in the frequency of *TP53* mutations^10^. We found that the most common point mutation in smokers (R158L) is less common in never-smokers (14/256 point mutations in smokers, vs. 0/57 in never smokers), although this difference was not significant (*p* = 0.15, χ^2^ test; Fig. 2D). This mutation has previously been shown to be more prevalent in lung cancers^10^ and is associated with changes in cell motility and drug sensitivity *in vitro*^11^. In summary, patients with NSCLC with a history of smoking had more frequent mutations in *TP53*, likely due to smoking-related mutational processes. Our Cox modeling results (Fig. 1) suggest that this increased *TP53* mutation burden is associated with increased risk of developing distant metastases after diagnosis.

Our work has several limitations. First, as our study retrospectively examined the effect of genomic alterations on patient outcome, differences in treatment or other factors associated with specific mutations (e.g., administration of targeted therapies to patients with *EGFR* mutations) made it difficult to isolate the effect of certain genomic changes. Additionally, our study is vulnerable to selection bias and to informative cohort entry^12,13^, since it only included patients who underwent primary tumor genomic sequencing, which is more likely to be performed in patients who later developed recurrent or progressive disease.

In summary, we found that *TP53* mutations are associated with distant recurrence in patients with NSCLC who were diagnosed with stage I-III disease. Our results suggest that *TP53* mutation status should be regularly tracked in all prospective adjuvant trials in early-stage NSCLC, so that the effect of this frequent mutation can be better understood. While previous clinical trials suggest that adjuvant therapy with cisplatin-based regimens does not improve survival in patients with early-stage *TP53*-mutant NSCLC relative to patients with *TP53*-wild type disease^14,15^, other therapies (e.g., immunotherapy) could provide a survival advantage to this population^16^. Given the potential for distant recurrence in this population, additional investigation of the optimal management strategy for patients with *TP53*-mutant NSCLC is warranted.

## Methods

### Participant eligibility and selection

Clinical and genomic data for 1,862 patients with NSCLC were collected as part of AACR Project GENIE (BPC NSCLC version 2.1) (Fig. 1A; Supplementary Table 1). Permission to access the data was granted by the AACR Project GENIE Biopharmaceutical Consortium publications committee. All patient data was anonymized before retrieval. The Dana-Farber/Harvard Cancer Center Institutional Review Board determined that this study did not constitute human subjects research, given its use of a previously collected, deidentified dataset. All research was performed in accordance with the Declaration of Helsinki. Data from patients with a NSCLC diagnosis of any stage and who received targeted genomic sequencing of a primary tumor and/or a metastasis biopsy at Dana-Farber Cancer Institute, Memorial Sloan-Kettering Cancer Center, or Vanderbilt-Ingram Cancer Center between 1/1/2014 and 12/31/2017, or at Princess Margaret Cancer Center (Toronto, CA) between 1/1/2014 and 12/31/2015 were collected in the BPC dataset. Additionally, the BPC study only included patients that were between 18 and 89 years of age at the time of sequencing and who were followed for at least two years after sequencing (or until death). For patients who had tumor sequencing performed on a research basis, informed consent for use of genomic and clinical data were obtained; for those who had sequencing performed on a standard of care clinical basis, data were collected under a waiver of informed consent at respective institutions. For this study, only patients with sequencing of at least one primary tumor sample were included, and only primary tumor sequencing data was used for all analyses. American Joint Committee on Cancer (AJCC) TNM tumor stage was determined in accordance with current guidelines at the time of diagnosis (AJCC guidelines version 6 or 7). Only patients with stage I-III disease at diagnosis were used for Cox proportional hazards modeling to study the association between primary tumor genomics, distant metastasis, and survival, while all patients (including patients with stage IV disease) were used to study the pattern of mutations that occur in the *TP53* gene in NSCLC.

### Clinical and genomic data collection

Targeted sequencing of primary tumor samples was performed using institution-specific clinical next-generation sequencing panels. The tumor sequencing panels used and variant calling pipeline for the AACR Project GENIE are as previously described^6^.

Imaging records and medical oncologists’ notes were curated according to the PRISSMM framework^17^ to determine when and where metastases appeared in each patient. Each radiologist report was reviewed to determine whether cancer was present and in which anatomical sites the tumor was found. These notes were used to determine the length of time from diagnosis of the primary tumor to the time at which disease was first observed at each distant site. The time to first distant metastasis was defined as the earliest time after diagnosis at which the patient had an extra-thoracic lymph node or organ metastasis, or a metastasis to the mediastinum, heart, or pleura. No patients in the analysis of association between primary tumor genomics and distant metastases had distant metastases at the time of diagnosis.

### Statistical analysis of time to new distant metastases

We used multivariable Cox proportional hazards models to test whether a priori defined static covariates were significantly associated with the development of new distant metastases after diagnosis in patients with stage I-III NSCLC. Six demographic and clinical covariates were included in each model: age at diagnosis, smoking history (current or former smoker vs. never smoker), sex, race, ethnicity, and stage (I, II, or III) at diagnosis. We also used primary tumor SNV/indel information for 5 genes (*TP53, KRAS, EGFR, BRAF, PIK3CA*) and copy number alteration data for 5 genes (*EGFR, PIK3CA, MET, KRAS, FGFR1*). Among mutations, only nonsynonymous point mutations, frameshift mutations, and splice site mutations were considered.

Multivariate Cox proportional hazards models for the time to first distant metastasis and for the time to bone, brain, and liver metastases were fit using the coxph function in the R survival package, version 3.2^18^, with right censoring at the date of death or last patient contact, such that the competing risk of death was addressed by analyzing the cause-specific hazard of distant metastasis. Wald test *p*-values for each covariate were pooled across all mutations/CNAs tested for each metastasis site and adjusted for multiple hypotheses^19,20^ using the Benjamini–Hochberg method, and covariates with adjusted *p*-value < 0.05 were considered significant. Confidence intervals for the hazard ratios were adjusted for multiple comparisons using the Bonferroni method.

### Statistical analysis of the effect of TP53 mutations on patient survival after NSCLC diagnosis

After observing that mutations in *TP53* were associated with increased risk of distant metastasis, multivariable Cox proportional hazards modeling was used to measure whether associations between primary tumor *TP53* mutation status were related to overall survival after diagnosis of stage I-III NSCLC. This model incorporated the demographic, clinical, and genomic covariates used in the time-to-metastasis models (age, sex, race, ethnicity, smoking history, stage at diagnosis, and 10 total mutation/copy number alteration variables). Risk set adjustment^21^ was not performed, since informative cohort entry has previously been demonstrated in clinico-genomic datasets^12,13^, and risk set adjustment could still yield biased results in the event of informative entry. Since this analysis was designed to specifically assess the effect of *TP53* mutations on patient survival, no correction for multiple hypotheses was performed.

## Supplementary Information

Supplementary Information 1.

Supplementary Information 2.

## Data Availability

Genomic and clinical data for the AACR Project GENIE BPC NSCLC cohort is publicly available at http://www.synapse.org/bpc.
