# Supplementary material for: Genomic analysis of early-stage lung cancer reveals a role for TP53 mutations in distant metastasis
Source: Sci Rep. 2022 Nov 9;12:19055. doi: 10.1038/s41598-022-21448-1 (PMC9646734; doi:10.1038/s41598-022-21448-1)
Supplement: Supplementary file 2 — Supplementary Information 2. [file 41598_2022_21448_MOESM2_ESM.pdf]

SUPPLEMENTARY INFORMATION FOR Genomic analysis of early-stage lung cancer reveals a role for *TP53* mutations in distant metastasis

Debra Van Egeren[#], Khushi Kohli[#], Jeremy L. Warner, Philippe L. Bedard, Gregory Riely, Eva Lepisto, Deborah Schrag, Michele LeNoue-Newton, Paul Catalano, Kenneth L. Kehl, and Franziska Michor[*], for the AACR Project GENIE Consortium

Demographic Profile	Number of Patients (n)	Percentage of Patients (%)
**Sex**		
Female	573	55.42%
Male	461	44.58%
**Race**		
White	866	83.75%
Black	52	5.03%
Chinese	46	4.45%
Other	20	1.93%
Unknown	30	2.90%
**Age**		
18-59	273	26.40%
60+	761	73.60%
**Smoking History**		
Has Smoking History	791	76.50%
No Smoking History	243	23.50%
**Ethnicity**		
Non-Spanish; non-Hispanic	997	96.42%
Spanish or Hispanic or Latino	37	3.58%
**Mutation Status**		
TP53 mutant	357	34.53%
KRAS mutant	249	24.08%
EGFR mutant	90	8.70%
BRAF mutant	46	4.45%
PIK3CA mutant	46	4.45%
**CNA status**		
EGFR amplified	73	7.06%
PIK3CA amplified	55	5.32%
MET amplified	50	4.84%
KRAS amplified	39	3.77%
FGFR1 amplified	38	3.68%
**Stage at Diagnosis**		
Stage I	419	40.52%
Stage II	133	12.86%
Stage III	189	18.28%
Stage IV	273	26.40%
Unknown	20	1.93%

Supplementary Table 1. Clinical, demographic, and genomic characteristics of the 1034 NSCLC patients used in this study. Patients diagnosed at stages I-III were used to assess the impact of genomic factors on the risk of developing new metastases, while patients diagnosed at any stage were used to determine where in the *TP53* gene mutations occurred in NSCLC patients.

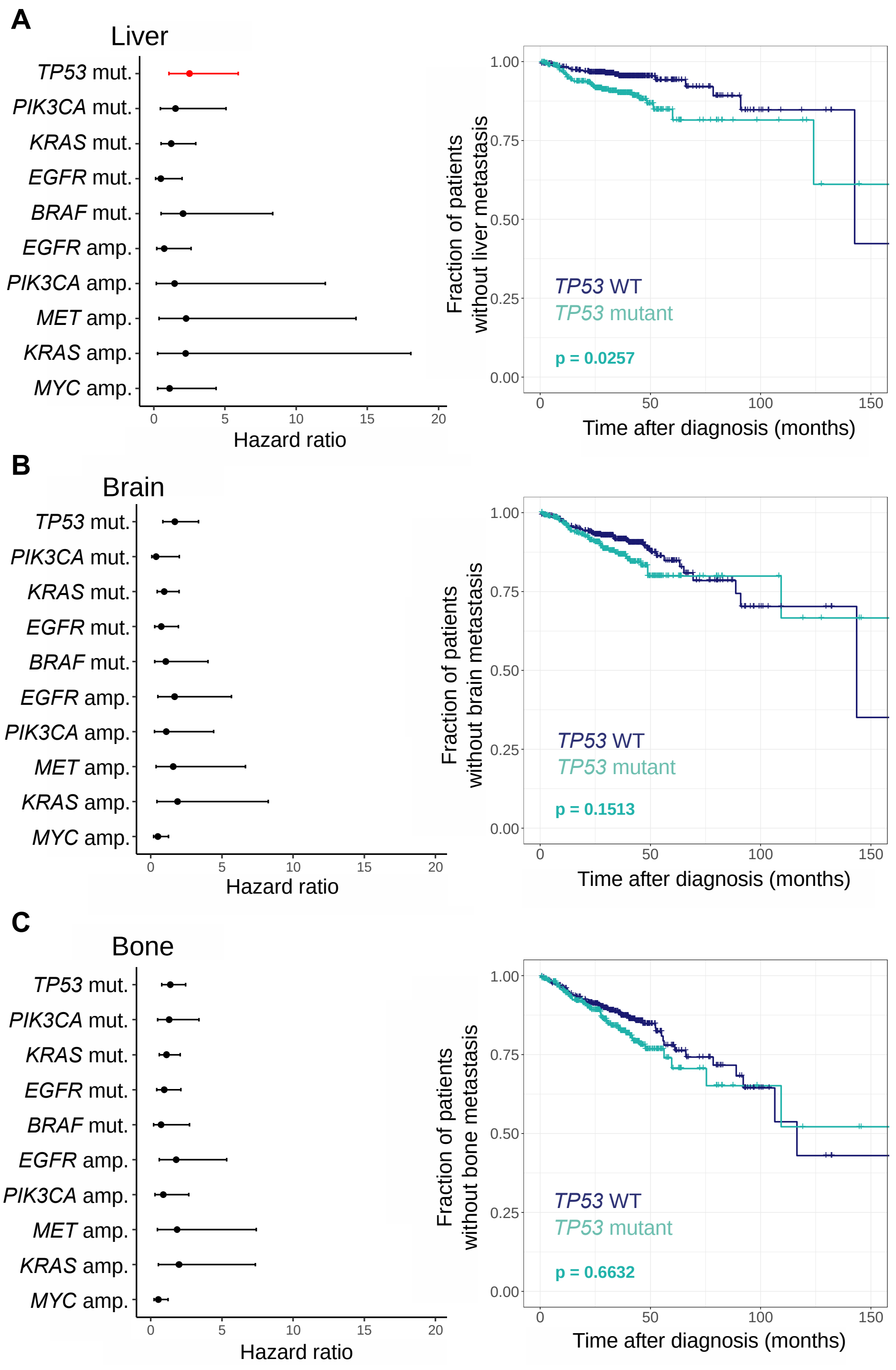

Supplementary Figure 1. ***TP53* mutations are significantly associated with the development of distant liver metastases after diagnosis in NSCLC, but not brain or bone metastases. A-C.** Left: forest plots depict the Cox regression hazard ratio of each mutation analyzed for liver, brain, and bone metastases, respectively. Significant results (α=0.05) are shown in red. Error bars are Bonferroni-adjusted 95% confidence intervals. Right: Kaplan-Meier plots depict the probability of developing new liver, brain, and bone metastases, respectively, stratified by *TP53* mutation status. The shaded regions shown in the Kaplan-Meier plots denote 95% confidence intervals. P-values denote the significance of each Cox hazard ratio.

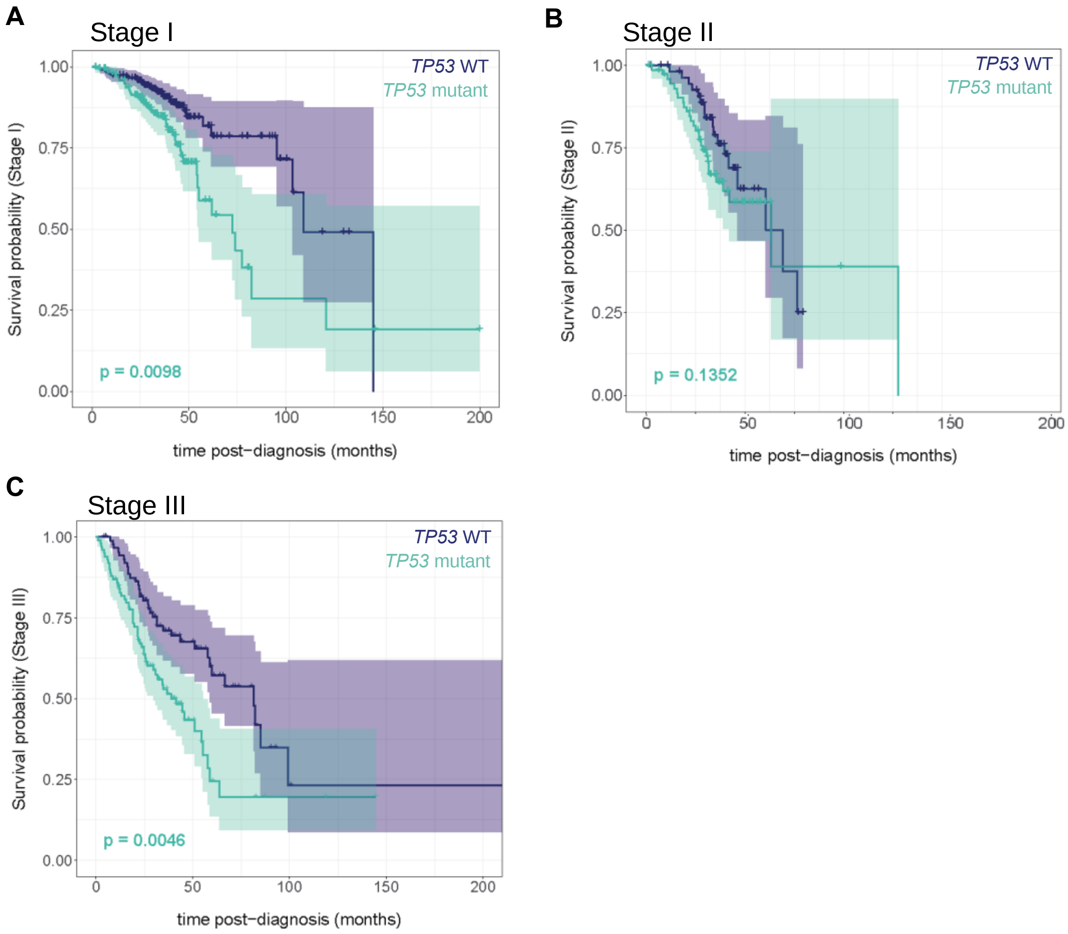

Supplementary Figure 2. *TP53* mutations are negatively associated with survival, particularly in early-stage patients. A-C. Kaplan-Meier curves showing NSCLC survival probability stratified by *TP53* mutation for patients diagnosed with Stage I, II, and III NSCLC, respectively. Colors for all panels denote primary tumor *TP53* mutation status (dark blue: *TP53* mutant, teal: *TP53* WT). Shaded regions denote 95% confidence intervals.
